# Computer Vision Based Automatic Recognition of Pointer Instruments: Data Set Optimization and Reading

**DOI:** 10.3390/e23030272

**Published:** 2021-02-25

**Authors:** Lu Wang, Peng Wang, Linhai Wu, Lijia Xu, Peng Huang, Zhiliang Kang

**Affiliations:** College of Mechanical and Electrical Engineering, Sichuan Agricultural University, Ya’an 625000, China; 201800656@stu.sicau.edu.cn (L.W.); 2019317016@stu.sicau.edu.cn (P.W.); 201800655@stu.sicau.edu.cn (L.W.); 10887@stu.sicau.edu.cn (L.X.); 14130@stu.sicau.edu.cn (P.H.)

**Keywords:** pointer instrumentation, image processing, object detection, K-fold cross-validation, Faster-RCNN

## Abstract

With the promotion of intelligent substations, more and more robots have been used in industrial sites. However, most of the meter reading methods are interfered with by the complex background environment, which makes it difficult to extract the meter area and pointer centerline, which is difficult to meet the actual needs of the substation. To solve the current problems of pointer meter reading for industrial use, this paper studies the automatic reading method of pointer instruments by putting forward the Faster Region-based Convolutional Network (Faster-RCNN) based object detection integrating with traditional computer vision. Firstly, the Faster-RCNN is used to detect the target instrument panel region. At the same time, the Poisson fusion method is proposed to expand the data set. The K-fold verification algorithm is used to optimize the quality of the data set, which solves the lack of quantity and low quality of the data set, and the accuracy of target detection is improved. Then, through some image processing methods, the image is preprocessed. Finally, the position of the centerline of the pointer is detected by the Hough transform, and the reading can be obtained. The evaluation of the algorithm performance shows that the method proposed in this paper is suitable for automatic reading of pointer meters in the substation environment, and provides a feasible idea for the target detection and reading of pointer meters.

## 1. Introduction

**Figure 1:**
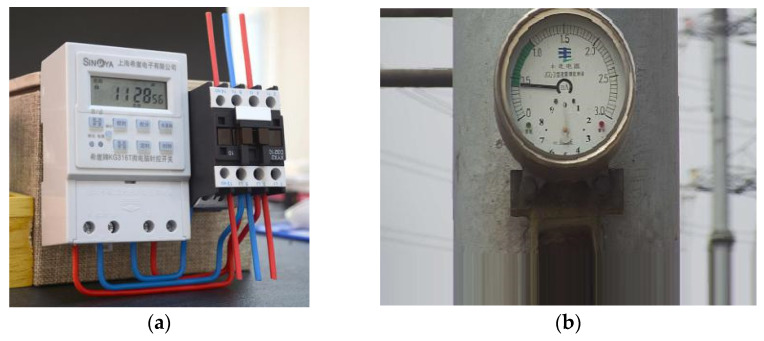
Working status of different instruments: (**a**) Digital display instrument; (**b**) pointer instrument.

There are many instruments in substations, such as pressure gauges, ammeters, oil temperature gauges, and so on. There are two types of current instruments, i.e., pointer type and digital type. Figure 1 shows the working conditions of pointer instruments and digital display instruments in substations.

Different working principles of such two types lead to different characteristics of them: the share of digital display instruments in the instrument industry is increasing for their advantages of high accuracy and convenient reading, while they are also not applicable to some occasions such as harsh oily or dusty environments, or this type of instrument won’t be applicable when the field instrument input variables change over fast [1]; In this regard, the pointer instrument has many advantages over the digital display instrument including simple structure, low price, strong anti-interference ability, dustproof, waterproof, anti-freeze, anti-interference, oil resistance, and so on [2].

Based on the above reasons, pointer instruments have been widely using in the industry, while recognition of their numerical value has always been a hot spot in the industrial study. In the early days of industrial development, the operators read the pointer instruments mainly by visual recognition, that is, manual interpretation, so it is inevitably subject to interferences caused by various artificial factors. Due to a large number of meters, the complex external environment, the dial observation angle, visual fatigue, the error caused by the observation distance and the deviation caused by the influence of light on the pointer artificial, and other factors, the recognition process is boring, troublesome and easily affected by subjective factors, which will inevitably cause inaccurate readings [1,2]. As technology develops, inspection robots have been introduced into more and more substations to replace manual inspections over recent years. Thereby the automation degree of substations has been greatly improved. The solution in this article is implemented offline. The robot takes photos and stores them, and then sends them to the computer for processing.

At present, there has been a lot of research on the automatic recognition of pointer meters. Peng et al. obtained pointer connection by converting Red, Green, Blue (RGB) space to Hue, Saturation, Value (HSV) space and utilizing color features to detect the beginning and end scales of oil level gauges. However, this method relies highly on environmental factors. Hua et al. [3] proposed a recognition algorithm for the readings of pointer instruments. It uses equipment images of the instrument to establish instrument templates and utilizes Scale-Invariant Feature Transform (SIFT), Oriented Fast and Rotated Brief (ORB), and other feature point detection algorithms, matching and extracting sub-images of the instrument dial area from the input image to realize instrument positioning. However, the template matching algorithm relies strictly on corner calibration, and corner detection is susceptible to deformation, occlusion, and other environmental factors, and a large number of false matches are likely to occur in complex scenes. To detect the instrument dial, Wei et al. [4] adopted Support Vector Machine (SVM) training, but the training accuracy of large sample data is not satisfactory [5]. Haoqiang et al. performed target detection over dial plate with the Single Shot multibox Detector (SSD) deep learning model to extract the meter area and remove irrelevant background [6]. The above methods are not ideal for object detection.

In recent years, the deep learning-based neural network method has been widely applied in the image processing of electric power meters. However, little research describes the data set. In the actual operation, the quality of the data set is critical to the accuracy of object detection. To ensure that the intelligent meter reading system forms a complex scene to obtain high-quality images of the dial area, this paper proposes to expand the data set with Poisson fusion algorithms to achieve its diversity, and then use the K-fold verification method to preprocess and optimize the training data aiming at fully training the model and suppressing the sample over-fitting caused by the insufficient quantity and uneven distribution. The utilization rate of the data set and the accuracy of model detection are greatly optimized. These data set optimization methods are unprecedented. On this basis, combined with the optimized classifier, the high-quality dial region image is extracted for image processing. The main contributions are as follows:

(1) Establishing a data set optimized by data fusion expansion and K-fold verification algorithm and getting it applied to industrial production, which optimized the data set quality and greatly reduced the workload of data set collection.
(2) An intelligent meter reading system whose accuracy rate up to 98.65% is obtained by utilizing Faster-RCNN and Hough transform straight line detection. The evaluation of the algorithm performance shows that the method proposed in this paper is suitable for the extraction of pointer meters in the substation environment, and a feasible thought for object detection and reading of pointer meters is provided thereof.

**Figure 2:**
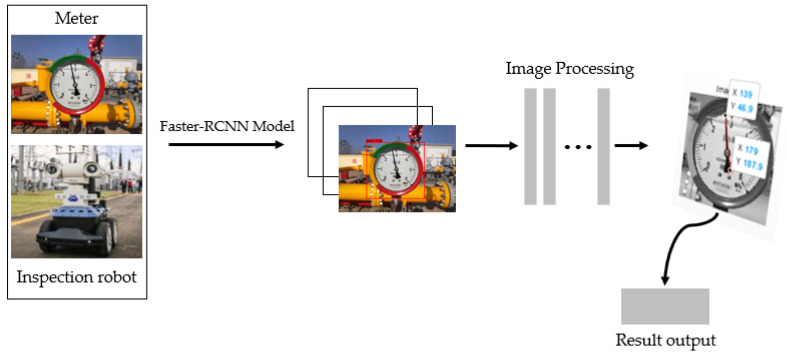
The overall flow of the solution in this paper is that: the image obtained by the robot is passed through the Faster-RCNN network to obtain the image of the dial area, and then preprocessed, the pointer position is detected by the Hough transform line, and finally, the indication is obtained.

The overall scheme of the method proposed in this paper is shown in Figure 2. All experiments are performed on Visual Studio 2019. Visual Studio 2019 is a product developed by American Microsoft Corporation.

This paper is organized into six sections, including the present one. Section 2 introduces the one-stage algorithm and the two-stage algorithm in deep learning compares the pros and cons of the two algorithms and explains the reasons why the Faster-RCNN algorithm is chosen. Section 3 describes the optimization of the data set and introduces how to improve the quantity and quality of data sets in detail. And the experimental results of the optimized data set are discussed therein. Section 4 explains the Faster-RCNN algorithm-based object detection network and the relevant network structure used in this paper. And it also compares and analyzes various classic classification network models. Section 5 elaborately describes dial image preprocessing and identifying the reading of pointer meter with Hough transform [7] and discusses the feasibility and practicability of the experimental results. Section 6 emphasizes some significant features of this scheme.

## 2. Deep Learning-Based Object Detection

### 2.1. Object Detection Algorithm

In the substation and factory environment, the accuracy of the reading of the pointer instrument is of primary importance to improving production efficiency and avoiding excessive losses. When an inspection robot shoots the images of a pointer instrument, it will inevitably include the complex background into the images. Accurately obtaining high-quality dial images from complex environmental backgrounds is the basis of accurate readings for the intelligent meter reading system. With regard to extraction and determination of shallow surface features of specific objects in the early development of object detection, Hough transforms [7,8,9], circle detection algorithm, and SIFT [10] feature matching algorithm are more taken among traditional computer vision methods. These two algorithms carry out detection based on certain features concluded from a large amount of research and summary work done by professionals, but this method has poor recognition accuracy, and the detection effect is quite limited. Aiming at this shortcoming, this paper uses a deep learning-based object detection algorithm, and by establishing a deep neural network model and using a large amount of data for training, the network model can more accurately identify the target from a complex background with high reliability and wider scope of adaptation.

The neural network-based object detection algorithms can be roughly divided into two-stage and one-stage. The two-stage detection algorithm divides the object detection process into two steps. First, it generates region proposals [11] relying on the Convolutional Neural Network (CNN to obtain the possible positions of the targets in the entire picture, and then it classifies and refines the proposed regions before carrying out subtle target recognition. The typical algorithms of the two-stage model include Region-based Convolutional Neural Network (R-CNN) [12,13], Fast Region-based Convolutional Neural Network (Fast R-CNN) [14], Faster R-CNN [15], and so on. One-stage does not require the region proposal stage. The position coordinates and category probability of the target can be generated through the CNN to obtain the position coordinates and corresponding confidence of the target directly from the picture. Typical algorithms of the one-stage model include You Only Look Once (YOLO) [16] and SSD [17].

### 2.2. Algorithm Selection

To select the best model, several commonly used models are tested, and the speed and accuracy are compared, as shown in Table 1. The Faster R-CNN algorithm has the best performance among the R-CNN series. This algorithm integrates the regional recommendation network and the convolutional neural network to classify and locate the target. Comparing with the other R-CNN series algorithms, it greatly reduces the time and space for training and testing, improves the detection speed while maintaining superiority over small object detection [12,13,14,15]. The YOLO series pursues detection speed at the expense of accuracy. The SSD algorithm strikes a balance between speed and accuracy, gaining a greater increase in speed at the expense of less decrease in accuracy [16,17]. However, in principle, the meter reading system in the substation does not allow false negatives, so this paper selects the Faster R-CNN model with higher detection accuracy and faster rate.

## 3. Preparation and Optimization of Data Set

The pictures in this paper were taken at a substation in Nanchong City, Sichuan Province. This data set was taken by Canon EOS90D. The camera comes from Canon Inc., Japan. The image resolution is 3472 × 2320. There are both long-distance shots and short-distance shots, and the shooting angles are both top-down and parallel. The data sets are dominated by top-down and short-distance shooting since it is more in line with the angular range of the inspection robot camera and is more practical. There is a great variety of pointer instruments in substations and factories, of which common types of instruments for measuring electrical quantities include ammeters, voltmeters, power meters, etc., while non-electrical quantities are mainly measured by pressure gauges, thermometers, and oil level gauges. To achieve better detection results, it is necessary to shoot tens of thousands of images in substations and factories for preparing a large number of data sets, but currently, there is no public instrument data set in substations, so we have to collect images by ourselves. This also results in a substantial increase in the workload of data set acquisition. Given the labor cost, this study obtained almost 1000 images through field shooting and from the Internet. To enrich the data set, the following process had been performed on the images for achieving high-quality data sets.

### 3.1. Data Set Processing

**Figure 3:**
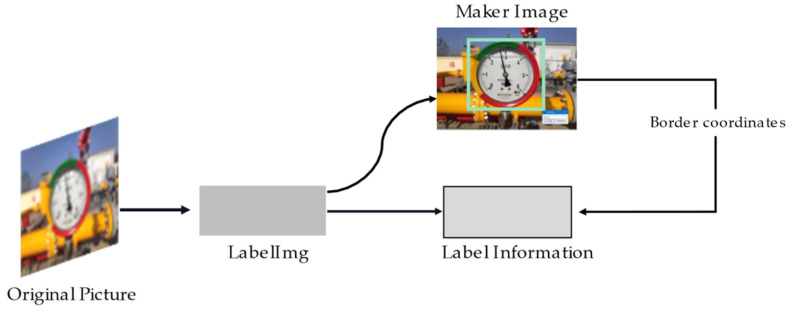
Preliminary meter image processing: Obtaining the label information of the dial position from original images.

Only a part of the region in any given image contains meters. As shown in Figure 3. The open-source software labelImg was employed to mark and generate corresponding label information, and then randomly cut such information according to the location of the meter.

### 3.2. Data Set Expansion

Because the shooting distance and the shooting time significantly affect the pixels and the quality of the images with the instrument on them, and the data set is not large enough, we expand the data set by randomly flipping and cropping the images. Two images of different objects were merged to obtain a higher recall rate. This method mainly merges training images with different targets through pixel-by-pixel blending, thus improving the generalization performance of image classification and object detection.

#### Data Fusion

The real captured image scenes are much more complicated than the cropped image contained in the data set. The background of the image may include complex objects that interfere with dial recognition, such as buildings, pipes, electric boxes, and electric poles. The complex background in the image causes a high false detection rate. To adapt to the complex background environment, it should compose images with different complex backgrounds by means of data fusion to add negative samples into the training data set and reduce false alarms by expanding the data set.

This paper uses the Poisson fusion method for image fusion when expanding data samples. The Poisson fusion method, as a well-known image editing algorithm, was proposed by R. Patrick Pérez in 2003 [18,19,20], which can achieve a more natural fusion effect and has been widely applied in image fusion and image restoration fields [21].

The Poisson fusion method regards the image fusion problem as a solution to the minimization problem of Formula (1).

(1) $$\underset{f}{\min}\iint {\left|\;\nabla f-v\;\right|}^{2},f{\left|{\;}_{\partial \Omega }=f^{*}\;\right|}_{\partial \Omega }$$

where Ω is the fusion area corresponding to the foreground image; ∂Ω is the boundary of such area; represents the fused image; $f^{*}$ represents fusing the known background image outside the fusion area; v is called guide field, and the gradient field of the original foreground image is taken. The minimization problem is to minimize the gap between the gradient field of the fusion area and the guide field while guaranteeing the boundary value of the fusion area is consistent with the background image, to preserve the gradient field of the original foreground image to the greatest extent. This problem can be transformed into the solution of Poisson’s equation with Dirichlet boundary conditions [22].

**Figure 4:**
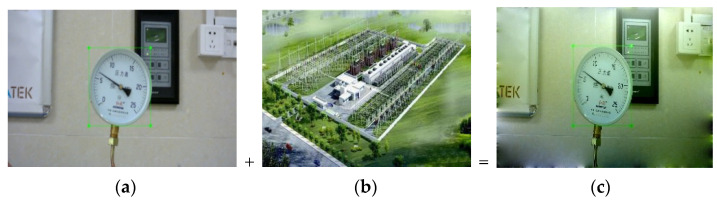
Composing example: (**a**) original image; (**b**) background image; (**c**) blended image.

Figure 4 shows a composing example. According to the gradient information of the source image and the boundary information of the target image, the image pixels within the composing area are reconstructed by means of interpolation. This method is based on Poisson image fusion to fuse background and dial samples to enrich the data set and form new samples.

It greatly reduced the workload of data acquisition and improved the object detection accuracy that the amount of data set was expanded through data fusion.

### 3.3. K-Fold Verification Algorithm

Overfitting, caused by an inadequate data set, is used in this paper. To improve the detection accuracy, this paper chooses the k-fold method [23] to preprocess the data set. With regard to this data set, the training set and the validation set are divided by 4:1. In this experiment, the value of k is 5, i.e., the training data is randomly divided into 5 portions, one of them is taken as the validation set and the other 4 portions are used as the training set. The original Faster-RCNN model is trained for 5 rounds in total, and we obtained the mean Average Precision (mAP) of 5 sets of model parameters. They are respectively used for testing the test set, and the original MobileNet V2 network framework is uniformly used in this experiment. Finally, the average of 5 test results is used as an evaluation of model accuracy. The test results of the 5 rounds of training in this experiment and the comparative experimental results of the final model evaluation are shown in Table 2 and Table 3.

By employing the K-fold verification data preprocessing method, it is significantly improved comparing with the original Faster-RCNN model. Moreover, the mAP of the model is increased by 2.76% after data preprocessing. This data set shows that the K-fold verification data preprocessing method has a good effect on inhibiting overfitting, which is beneficial to optimize model parameters and improve model detection performance.

## 4. Object Detection Results and Experimental Process

### 4.1. Faster-RCNN Based Dial Object Detection

**Figure 5:**
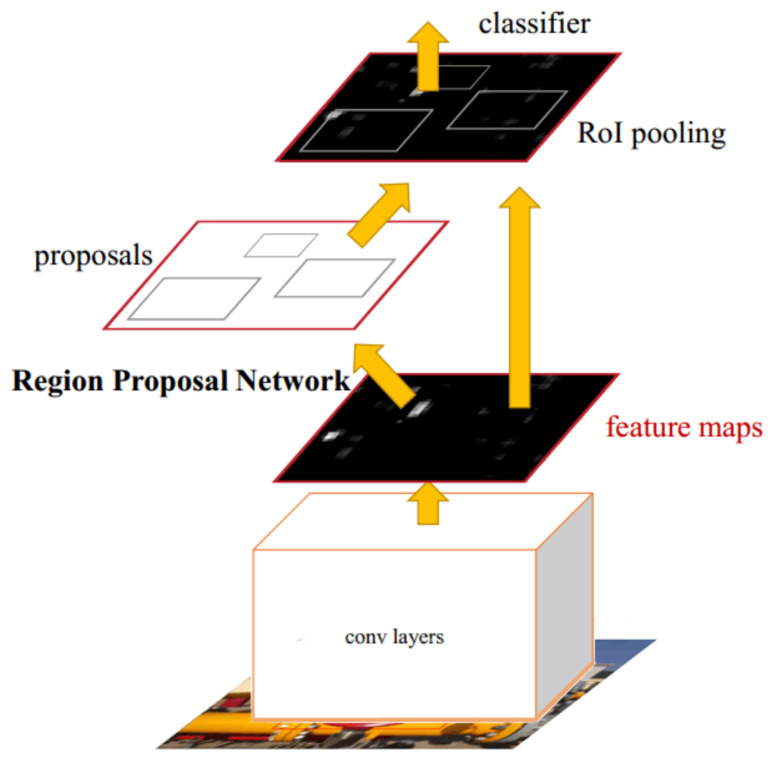
RPN-based Faster-RCNN network structure.

The RPN-based Faster-RCNN network structure is shown in Figure 5. The algorithm model sets an N × N sliding window on the feature map of the input image, and each sliding window can be mapped to a fixed-dimensional feature. If Anchors set k different aspect ratios, each window will correspond to k candidate regions of different sizes, and then their features will be sent to two fully connected layers for category prediction and border regression. The output dimension of the classification layer is 2k, which represents the probability that the candidate area contains the target or background; the output dimension of the border regression layer is 4k, which represents the location information of the candidate area. The RPN network can be trained separately for effectively reducing the time to generate the feature map of the candidate region. In this way, the training speed of Faster R-CNN is greatly improved over Fast R-CNN.

### 4.2. The Core Feature Extracting Network Commonly Used by the Faster R-CNN Algorithm

#### 4.2.1. ResNet

Residual Network(ResNet) [24], as a residual network, can be understood as a sub-network, which can form a deep network after stacking. As a general rule, the deeper the network, the more information obtained and the richer features. However, the deepening of the network did not improve the optimization effect, and the accuracy of the test data and training data was decreased. To solve the problem of gradient explosion and gradient disappearance caused by network deepening, four Chinese Scholars including Kaiming He of Microsoft Research proposed ResNet. They successfully trained a neural network with a depth of up to 152 layers on the image network data set, and only a 3.57% error rate in the top 5. This result won first place in the ILSVRC 2015 Classification Task. Besides, the parameter quantity is lower than Visual Geometry Group Network(VGGNet), and the effect is outstanding.

#### 4.2.2. VGGNet

The VGG [25] network model, as a deep learning convolutional neural network model, was proposed by the Visual Geometry Group of Oxford. VGGNet explored the relationship between the depth and the performance of a convolutional neural network. In short, VGG successfully builds a convolutional neural network with a depth of 16–19 layers by repeatedly stacking 3 × 3 convolution kernels and 2 × 2 pooling kernels. An improvement of VGG16 compared to Alex Krizhevsky Network(AlexNet) [26] is to use several consecutive 3 × 3 convolution kernels to replace the larger convolution kernels in AlexNet. In this way, it ensures that improving the network depth under the condition of the same receptive field can improve the neural network result to a certain extent. VGGNet won second place in the ILSVRC 2014 competition classification project and first place in the positioning project, with an error rate of 7.5% on the top 5. At present, VGGNet is also widely used in extracting image features.

#### 4.2.3. MobileNet

MobileNet [27] was proposed by Google in 2017. It is a lightweight CNN neural network focusing on mobile devices and embedded devices, and it quickly derived three versions of v1, v2, and v3. The main work of MobileNet is to replace the past standard convolution with depth-level separable convolution to solve the problem of computational efficiency and parameter amount of convolutional network. Deep convolution applies each convolution kernel to each channel, and 1 × 1 convolution is used to combine the output of channel convolution. Compared with the traditional CNN network, it greatly reduces model parameters and computation under the premise of a small reduction in accuracy.

#### 4.2.4. The Classifier Used in this Article

**Figure 6:**
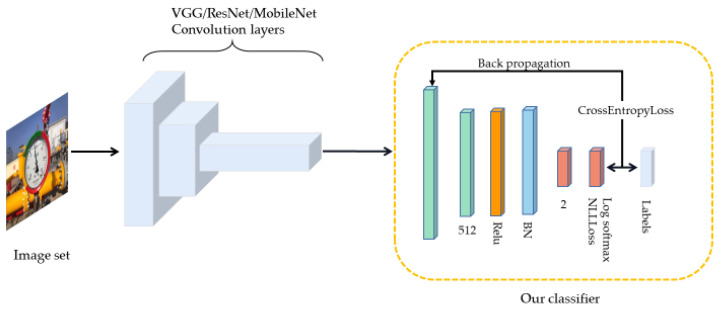
Classification network frame: integrating the convolutional layer of other classification networks after optimizing based on the original classifier.

The mainframe of the classifier used in this paper is shown in Figure 6. Combined with the network structure in Table 4, the classifier has only one hidden layer containing 512 dimensions, which is then activated by the ReLu activation function. Afterward, it outputs in two dimensions according to the number of classes in. To prevent over-fitting, convergence is accelerated for increasing the training speed and improving the object detection accuracy. The Batch Normalization (BN) layer is added and the Dropout layer is discarded. and process the final output into a pile to evaluate the accuracy of the model. The classifier uses CrossEntropyLoss [28] as the loss function. In the final output, the loss function has integrated the Log Softmax layer and the NLLLoss layer [29] to reduce computation. This loss is helpful for object detection problems.

### 4.3. Faster-RCNN Algorithm Results Based on Different Frames

#### 4.3.1. Experimental Environment

The experimental platform is configured with Intel Core CPU i7-10700k, 3.80 GHz CPU, 10 GB NVIDIA GeForce RTX3080 GPU, 32 GB DDR4 2666 Mhz memory, 1 TB Samsung 970 solid-state drive, and the operating system is Windows 10. The experimental equipment comes from China Lenovo.

#### 4.3.2. Evaluation Parameters

IoU represents the overlapping degree between the generated candidate bound and the ground truth bound, i.e., the ratio of their intersection and union. The larger the value, the higher the correlation. The ideal situation is complete overlapping, i.e., the ratio is 1. The specific formula is as follows:

(2) $$IoU=\frac{area\left(C\right)\cap area\left(G\right)}{area\left(C\right)\cup area\left(G\right)}$$

In Formula (2), C represents the candidate boundary, and the candidate bound is generated by the object detection model. G represents the actual labeling truth bound. The most common threshold is 0.5—if IoU > 0.5, it will be deemed as correct detection, otherwise it will be deemed as a wrong detection. For those objects that are deemed as correct predictions, they will be used to evaluate the overall accuracy of the final model.

The cross-entropy loss function is used to estimate the gap between the output of the model and the true value, and to guide model optimization. We usually use the Sigmoid function to compress the output of the model to the interval (0,1). $\hat{y_{i}}\in \left(0,1\right)$ is used to represent the probability that the given input $\hat{x_{i}}$ model is judged to be a positive class. The detailed formula is as follows:

(3) $$p(y_{i}=1|xi)=\hat{y_{i}}$$

(4) $$p\left(y_{i}=0|x_{i}\right)=1-\hat{y_{i}}$$

(5) $$p\left(y_{i}|x_{i}\right)={\hat{y_{i}}}^{y_{i}}{\left(1-\hat{y_{i}}\right)}^{1-yi}$$

(6) $$L\left(x,y\right)=\overset{N}{\underset{i=1}{\prod }}{\left(\hat{y_{i}}\right)}^{y_{i}}{\left(1-\hat{y_{i}}\right)}^{1-y_{i}}$$

(7) $$NLL\left(x,y\right)=-\overset{N}{\underset{i=1}{\sum }}y_{i}\log\left(\hat{y_{i}}\right)log\left(1-\hat{y_{i}}\right)$$

Formula (7) represents the output of the cross-entropy loss function, and loss is used to represent the cross-entropy loss function below. The other evaluation index parameters are defined as the following formulas:

(8) $$P=\frac{N\left(TurePositives\right)}{N\left(TotalObjects\right)}$$

(9) $$AP=\frac{\sum Precisions}{N\left(TotalImages\right)}$$

(10) $$mAP=\frac{\sum AP}{N\left(AP\right)}$$

In Formulas (8)–(10), P represents the prediction accuracy of each image; AP represents the overall average accuracy of all data sets; mAP is the mean average precision evaluated from multiple verification sets for use as an index for measuring accuracy in object detection; N represents the number of element types in brackets.

#### 4.3.3. Experimental Results

**Figure 7:**
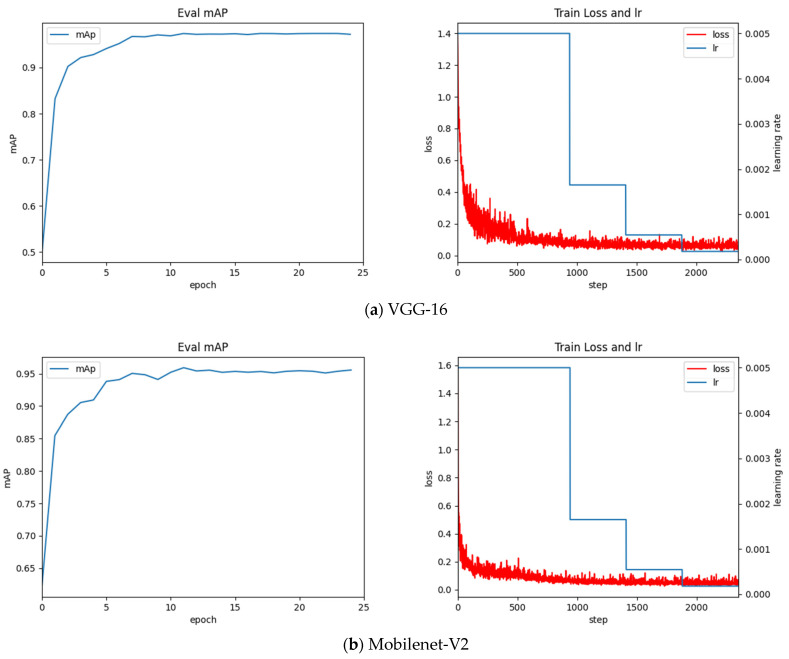

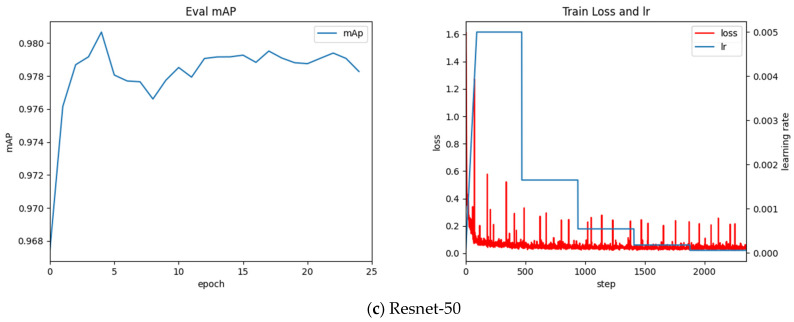
The left figure reflects the change of mAP (mean Average Precision) value in the training process, and the right figure shows the change of Faster-RCNN total loss and learning rate (lr) in the training process. Lr in Figure 7 represents the learning rate (Learning rate), as an important hyperparameter in supervised learning and deep learning, determines whether the objective function can converge to a local minimum and when to converge to the minimum. A proper learning rate can make the objective function converge to a local minimum in a proper time.

The detection algorithm is developed based on the deep learning language framework Torch. To achieve the best results, adjust the training hyperparameters for this data set, the training parameters of the Faster-RCNN algorithm are shown in Table 5. The training process is shown in Figure 7.

Based on the observations in Figure 7, we can draw the following conclusions:

- Through the above experiment, observe the above training process diagram, the best mAP value for the classification network VGG-16 is 97.49%, the best mAP value for the Resnet-50 network is 98.20%, and the best mAP value for the MobileNet-V2 network is 95.46%.
- Resnet-50 performs poorly in global optimization. Its accuracy in the latter period is high, but the final convergence effect is not ideal. The oscillation still exists under the optimized classifier in the later stage, and the experiment still fluctuates greatly.
- MobileNet-V2’s early oscillation is more serious, but the later convergence effect is favorable. Given its low mAP value, it is discarded.
- With regard to the data of this sample, the VGG-16 network finally converges well, and its performance in the classifier is also ideal. Experiments under the optimized classifier show good accuracy and convergence. It is proved that the global optimization of VGG-16 is the best classification network, and its mAP value converges to 0.974.

VGG-16 was selected as the model for object detection in this article because the combination of the convolutional layer of VGG-16 and our optimized classifier achieved favorable accuracy and the best convergence.

## 5. Processing and Analysis

The dial area is extracted from the complex background with the Faster-RCNN algorithm, and then the centerline of the pointer is accurately extracted with the corresponding image processing algorithm. The main content of this part is as follows: First, perform preprocessing such as binarization and image noise reduction on the dial image, then extract the centerline of the indicator pointer with the Hough line detection algorithm, and finally, obtain the pointer reading according to the deflection angle of the pointer.

### 5.1. Image Preprocessing

**Figure 8:**
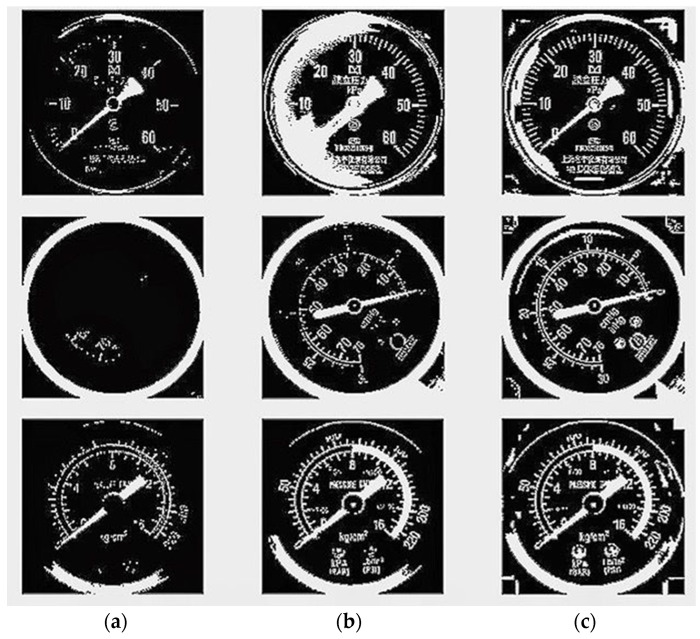
Comparison of binarization methods: (**a**) average grayscale method; (**b**) maximum between-class variance method; (**c**) maximum between-class variance local binarization; The average grayscale method cannot favorably reserve dial information. The maximum between-class variance method performs poorly in poor lighting conditions, so it is not practical in actual scenes. While the maximum between-class variance local binarization balances the integrity of the information and the reduction of the influence of lighting conditions.

**Figure 9:**
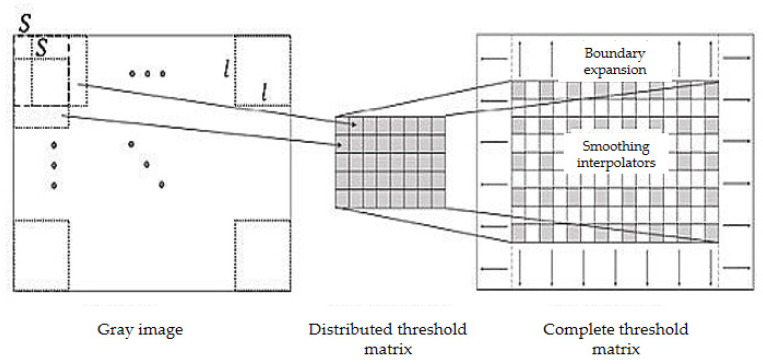
Represents local binarization of local blocks and moving step length.

To extract pointer information from the picture, it first has to gray the cropped color instrument image to obtain the gray image and get it binarized. Commonly used binarization algorithms include the average grayscale method, maximum between-class variance method, etc. [30]. Comparing the two methods, the average grayscale method has a lower calculation burden, while the maximum between-class variance method can better preserve the image information. According to experiments, for images with uneven brightness, using the maximum between-class variance method cannot balance the details of the brighter and darker parts of the image. Therefore, the local binarization method should be used to preserve the details of the image. Figure 8 shows the effects of different binarization methods. As shown in Figure 9, a local block of 1 square side length is selected from the image, and translated as per step size S to form a series of local blocks. In each local block, the result obtained by the best-performing maximum between-class variance method is taken as the binarization threshold, and each threshold is recorded to form a distribution threshold matrix. When local binarization is carried out with the average gray level method, it is greatly affected by the choice of local block size and the moving step length, and the parameter adaptation range is narrow. However, the effect is stable when local binarization is carried out with the maximum between-class variance. Therefore, this paper adopts the local binarization of maximum between-class variance. In the distribution threshold matrix, the brightness of some local blocks may be exceedingly bright or dark making the threshold deviate far from the average gray value, which causes abnormal spots in the binarized image. To reduce such influence, median filtering is performed on the distribution threshold matrix. The median filtered distribution threshold matrix is smoothly interpolated and boundary expanded to form a complete threshold matrix on the same dimension with the gray image. Binarizing the grayscale image according to the complete threshold matrix, the experimental results show good results.

**Figure 10:**
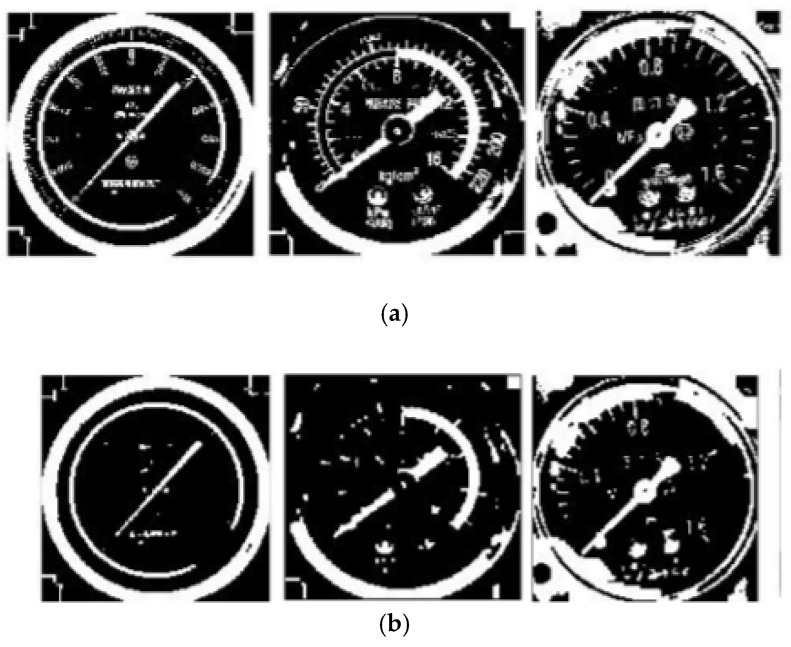
The binarization image and the open-close treated image are compared: (**a**) is the original image of the binarization; (**b**) the image after the opening and closing processing.

There may be noise or line breaking on the binarized image due to image quality or interference in the acquisition process, and the image after the meter pointer binarization is thicker but easy to have burrs. To eliminate thinner lines and burrs, smooth the boundary of the thick line, this paper performs image opening and closing processing on the binary image [31] to improve the accuracy of the subsequent Hough transform line detection. Figure 10 shows the effect of image open-close operations.

### 5.2. Pointer Extraction

**Figure 11:**
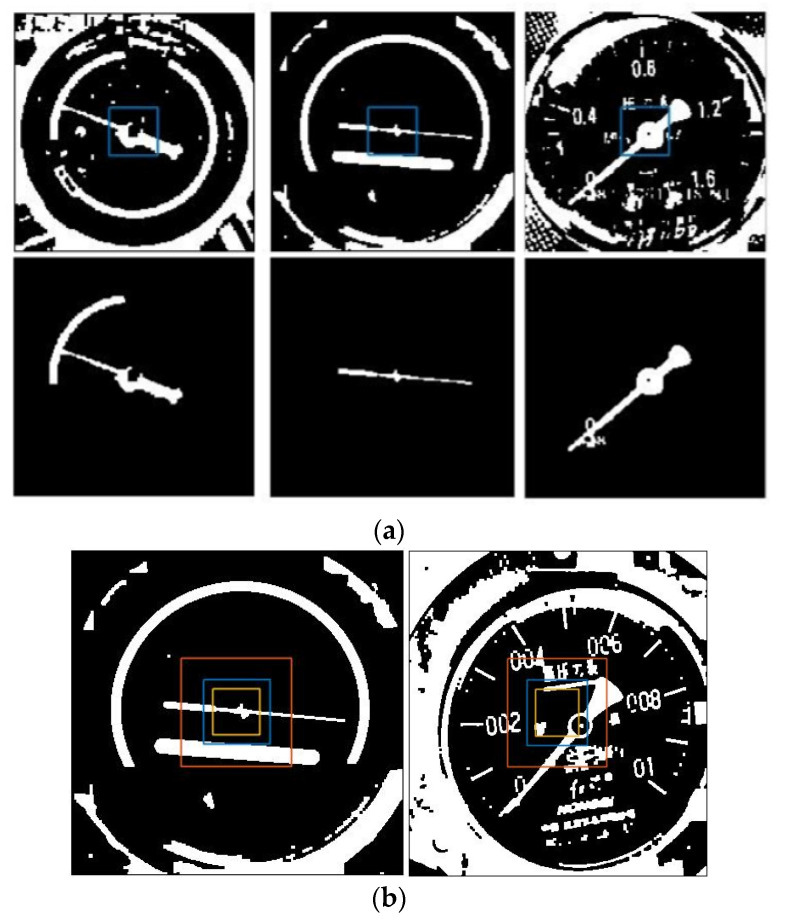
Extract pointer image: (**a**) Search image for the connected domain with the standard of 5 × 5 grid, and the central grid and the connected domain starting from the central grid is shown; (**b**) The comparison of the central grid with different specifications; interference graphics are easily introduced if the central grid is too large (3 × 3), and it cannot overcome the pointer missed detection problem caused by the deviation of the pointer position from the image center caused by the inaccurate object detection if it is too small (7 × 7).

The purpose of this article is to detect the pointer. When the meter can be accurately captured in the aforementioned object detection, the pointer will be in the middle of the binarized image. As shown in Figure 11. Divide the image into 5 × 5 divisions, and start from the central grid to find the connected domain [32] and delete the other images.

**Figure 12:**
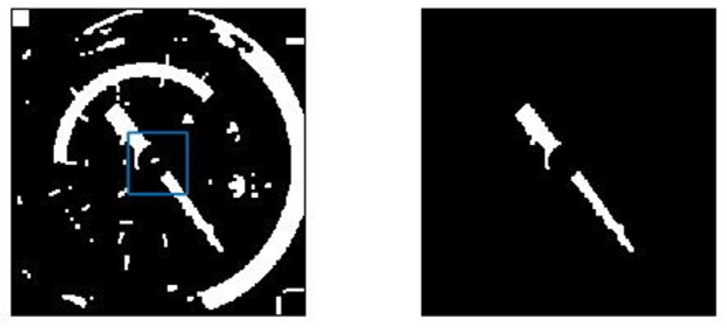
The situation of the broken pointer.

**Figure 13:**
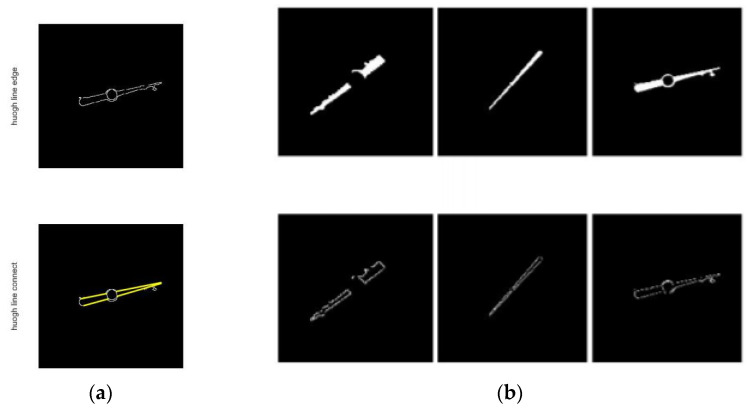
Line detection: (**a**) Perform boundary extraction on the filtered images of the connected domain; (**b**) Use Hough transform for straight line detection.

**Figure 14:**
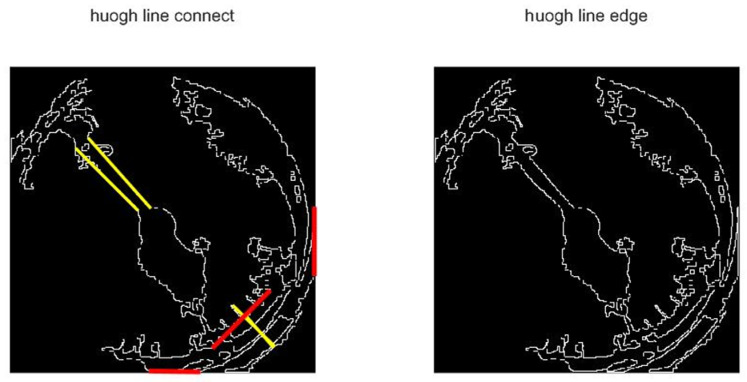
Straight-line filtering diagram: The extension of the yellow straight line passes through the central grid, while the extension of the red straight line does not pass through the central grid.

The central grid may not only retain the pointer image, but also other small spots. We also found in experiments that, as shown in Figure 12, the pointer image is often broken into two segments due to the brightness of the fixed axis is different from that of the pointer, thus the remaining connected domains should be filtered. Sort the connected domains by the area in descending order, keep the part with the largest area, and compare the ratio of the second-largest connected domain to the largest connected domain. If the area ratio is greater than 1/3, keep it, otherwise delete it together with the other connected domains. When there is only the pointer skeleton as shown in Figure 13, straight-line detection is performed on the pointer. As the connected domain of the pointer may contain other graphs, some interference lines may be obtained in the Hough transform line detection. As shown in Figure 14, the lines obtained by the Hough transform should be filtered. In case the object detection is accurate enough, the straight-line boundary will inevitably pass through the central grid of the 5 × 5 grid, so only straight lines that meet this condition are retained.

**Figure 15:**
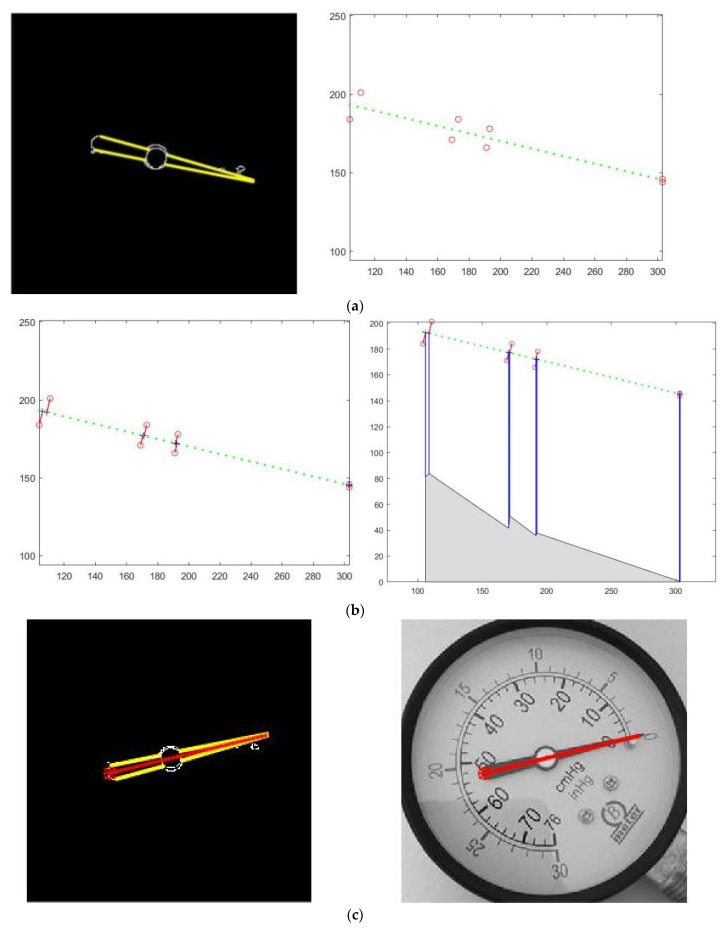

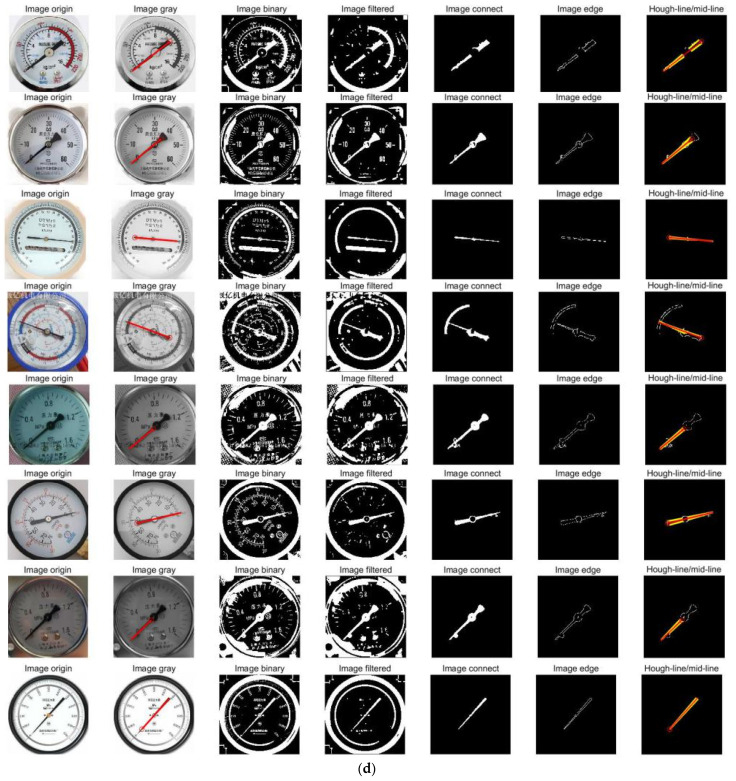
Pointer centerline extraction effect: (**a**) The linear fitting effect of the boundary line; (**b**) the vertical distance from the point on the boundary line to the fitted line, and the horizontal distribution of its square value; (**c**) the detected pointer center coincides with the pointer in the dial image when it is mapped to the original dial image; (**d**) shows the overall processing flow of extracting the final pointer centerline from the original dial image.

Put all the endpoints of all conforming straight lines together for linear fitting. Figure 15 shows the linear fitting effect of the endpoints of the straight line. The range limited by the leftmost and rightmost abscissas in the endpoints is used as the defined interval of the fitted straight line for indicating the pointer position. And the pointer center line is mapped to the original dial to observe the extraction effect of the pointer centerline.

The image processing algorithm is tested. The image processing algorithm has a large calculation burden, and the average time from image input to result output is 0.85 s.

### 5.3. Image Reading

After extracting the centerline of the pointer, the reading is obtained according to the deflection angle of the centerline. In this paper, the commonly used thermometers in substations are selected as the test objects, and 1/2 positive and 1/2 negative samples are selected as the test set. Table 6 shows the comparison of manual readings, existing research methods [3,4] readings, and readings obtained by the method used in this article. Since the identification process of different pointer instruments is similar, this conclusion can be applied to other types of instruments.

To reduce the error of manual readings, we used the average result of readings of five testers. The measuring range of the test instrument is 120 °C. It can be seen from Table 6 that the method proposed in this paper is basically consistent with the manual reading result, with an average error of 1.354% and a maximum relative error of 3.325%. The error of this scheme is obviously smaller than that of the other two methods. It proves that the recognition system proposed in this paper is more accurate.

## 6. Summary

With regard to recognition of pointer instrument reading, this paper abandons traditional feature point detection algorithms with low robust such as SIFT and ORB but introduces deep learning-based Faster R-CNN model and improved accuracy. Aiming at the number of the data set, we expand the data set with the method of Poisson fusion and use the k-fold cross-validation to preprocess the data set to improve the quality of it to optimize the model parameters. As far as we know, this work is applied in the indicator reading recognition area for the first time. For this data set, we designed a classifier and get it integrated with the convolutional layers of VGG-16, ResNet-50, and MobileNet V2 for experiments. According to the experimental results, the classifier performed well, with VGG-16 achieving the best performance with an average prediction accuracy of 97.49%. Finally, the position of the pointer of the meter is detected by image processing methods such as Hough transform, and finally, the reading of the pointer meter is obtained. The average relative error of the pointer angle obtained by this scheme is no more than 1.354%. The experimental results prove that the accuracy and stability of the detection and recognition system are suitable for practical application.

## Figures and Tables

**Table 1:** Test results of five models.

Model	R-CNN	Fast-RCNN	Faster-RCNN	YOLO	SSD
mAP (%)	63.20	70.21	90.27	81.32	79.20
Time (s)	22.1	4.1	0.69	0.48	0.57

**Table 2:** K-fold verification test results.

Target Detection	Epoch1 (%)	Epoch2 (%)	Epoch3 (%)	Epoch4 (%)	Epoch5 (%)
mAP	95.45	95.35	95.21	95.61	95.52

**Table 3:** Comparison of 5-fold data preprocessing based experimental results.

Target Detection	mAP (Faster-RCNN)	mAP (K-fold)
mAP	92.67	95.43

**Table 4:** Classification grid structure mentioned in the article. This article uses three classic classification networks. The X in conv X refers to the internal structure of the convolutional layer.

	VGG	ResNet	MobileNet	
	16-layer	50-layer	16-layer	
Conv1_×	3×3.64	7×7.64 stride 2	Type/stride	Filter Shape
	3×3.64		Conv/s2	3×3×3×32
	3×3maxpool	3×3maxpool	Conv dw/s1	3×3×32 dw
	stride 2	stride 2	Conv/s1	1×1×32×64
Conv2_×	3×3.128	$\left[\begin{array}{c} 1\times 1.64\\ 3\times 3.64\\ 1\times 1.256 \end{array}\right]\times 3$	Conv dw/s2	3×3×64 dw
	3×3.128		Conv/s1	1×1×64×128
	3×3maxpool,		Conv dw/s1	3×3×128 dw
	stride 2		Conv/s1	1×1×128×128
Conv3_×	3×3.256	$\left[\begin{array}{c} 1\times 1.128\\ 3\times 3.128\\ 1\times 1.512 \end{array}\right]\times 4$	Conv dw/s2	3×3×128 dw
	3×3.256		Conv/s1	1×1×128×256
	3×3.256		Conv dw/s1	3×3×256 dw
	3×3maxpool,		Conv/s1	1×1×256×256
	stride 2		Conv dw/s2	3×3×256 dw
Conv4_×	3×3.512	$\left[\begin{array}{c} 1\times 1.256\\ 3\times 3.256\\ 1\times 1.1024 \end{array}\right]\times 6$	Conv/s1	1×1×256×512
	3×3.512		$5\times \begin{array}{c} \mathrm{Conv}\;\mathrm{dw}/s1\\ \mathrm{Con}/s1 \end{array}$	3×3×512 dw 1×1×512×512
	3×3.512		Conv dw/s2	3×3×512 dw
	3×3maxpool,		Conv/s1	1×1×512×1024
	stride 2		Conv dw/s2	3×3×64 dw
Conv5_×	3×3.512	$\left[\begin{array}{c} 1\times 1.512\\ 3\times 3.512\\ 1\times 1.2048 \end{array}\right]\times 3$	Conv/s1	1×1×1024×1024
	3×3.512		Classificatin layer	fc 1000 softmax
	3×3.512			
	3×3maxpool,			
	stride 2			
Classification layer	fc 4906			
	fc 4906	fc 1000		
	fc 1000	softmax		
	softmax			

**Table 5:** Training hyperparameters.

	Learning Rate	Batch Size	Maximum Number of Epochs	Iterations Per Epoch	Momentum	Decay
Faster-RCNN	0.005	4	25	50	0.9	0.0005

**Table 6:** Test results of different methods for reading recognition.

Number	Human Vision	Method in [3]	Relative Error (%)	Method in [4]	Relative Error (%)	Our Method	Relative Error (%)
1	41.121	39.140	1.651	39.564	1.297	40.481	0.533
2	38.725	33.548	4.314	34.176	3.790	35.952	2.310
3	30.710	35.954	4.370	25.354	4.463	26.720	3.325
4	47.601	42.364	4.364	42.689	4.093	43.894	3.089
5	39.399	35.021	3.648	35.637	3.135	36.746	2.211
6	43.054	40.364	2.242	40.854	1.833	41.946	0.923
7	36.732	37.962	1.025	35.086	1.372	36.333	0.332
8	34.137	31.541	2.163	31.877	1.883	32.091	1.705
9	57.380	55.100	1.900	59.324	1.620	58.273	0.744
10	46.332	44.326	1.672	44.638	1.411	45.624	0.590
11	54.462	53.920	0.452	53.214	1.040	54.237	0.187
12	86.906	89.785	2.399	90.102	2.663	84.785	1.762
13	41.987	39.542	2.038	39.563	2.020	40.069	1.592
14	33.205	35.248	1.702	36.420	2.679	34.350	0.954
15	36.888	35.147	1.451	34.695	1.827	37.699	0.682
16	74.691	73.210	1.234	72.913	1.481	73.645	0.871
17	40.342	35.124	4.348	36.451	3.242	37.250	2.576
18	58.933	58.322	0.509	58.360	0.477	58.485	0.373
19	40.986	36.853	3.444	37.021	3.304	38.279	2.255
20	41.248	40.985	0.219	40.830	0.348	41.164	0.070
Mean	/	/	2.257	/	2.199	44.905	1.354

## Data Availability

Publicly available datasets were analyzed in this study. This data can be found here: [https://www.kaggle.com/wl13980027641/automatic-recognition-of-pointer-instruments, accessed on 24 February 2021].
